# Lutetium-177 Labelled PSMA Targeted Therapy in Advanced Prostate Cancer: Current Status and Future Perspectives

**DOI:** 10.3390/cancers13153715

**Published:** 2021-07-23

**Authors:** Konstantin Egon Seifert, Robert Seifert, Katharina Kessel, Wolfgang Roll, Katrin Schlack, Martin Boegemann, Kambiz Rahbar

**Affiliations:** 1 Department of Urology, University Hospital Muenster, 48149 Muenster, Germany; konstantin.seitzer@ukmuenster.de (K.E.S.); katrin.schlack@ukmuenster.de (K.S.); Martin.Boegemann@ukmuenster.de (M.B.); 2 Department of Nuclear Medicine, University Hospital Muenster, 48149 Muenster, Germany; robert.seifert@uni-muenster.de (R.S.); katharina.kessel@ukmuenster.de (K.K.); wolfgang.roll@ukmuenster.de (W.R.); 3 Department of Nuclear Medicine, University Hospital Essen, 45147 Essen, Germany

**Keywords:** prostate cancer, castration-resistant, prostate-specific membrane antigen, radioligand therapy, lutetium

## Abstract

**Simple Summary:**

For patients with advanced prostate cancer, many different treatment options are available. One option is the radioligand therapy with ^177^Lutetium labelled prostate-specific membrane antigen (Lu-PSMA). This treatment showed good results in previous studies with only some non-severe side effects. This review offers a short overview about the application, current standings and the future perspective of the radioligand therapy with Lu-PSMA. An approval of this therapy is awaited within 2021.

**Abstract:**

Patients suffering from metastatic castration-resistant prostate cancer (mCRPC) have a poor prognosis. As a further treatment option ^177^Lutetium (Lu) prostate-specific membrane antigen (PSMA) radioligand therapy gained a significant interest of many investigators. Several publications showed great response and prolonged survival with limited adverse events. However, to this point, it still remains unclear which patients benefit the most from ^177^Lu-PSMA therapy, and how to improve the treatment regimen to achieve best outcome while minimizing potential adverse events. The efficacy for mCRPC patients is a given fact, and with the newly published results of the VISION trial its approval is only a matter of time. Recently, investigators started to focus on treating prostate cancer patients in earlier disease stages and in combination with other compounds. This review gives a brief overview of the current state and the future perspectives of ^177^Lu labelled PSMA radioligand therapy.

## 1. Introduction

Metastatic castration-resistant prostate cancer (mCRPC) is a challenging situation for clinicians. Fast progression, short survival, and various treatment options make it difficult to handle. Numerous researchers around the world focus and work on optimizing treatment regimens, and try to develop new (radio)pharmaceuticals. In the last decade, Prostate-specific membrane antigen (PSMA) radioligand therapy (RLT) gained prominence in diagnosing and treating advanced prostate cancer. PSMA, also known as glutamate carboxypeptidase II or folate hydrolase I, is a membrane bound protein on the epithelial cells of the prostate. The name implies a specificity to the prostate gland. However, PSMA is also expressed in other sites such as the kidney, salivary gland, lacrimal gland, duodenal mucosa, and in other neoplastic tissue apart from prostate cancer [1,2]. Compared to physiological uptake, PSMA is generally overexpressed and upregulated in prostate cancer cells, making it interesting for targeted imaging and therapy [1,3,4]. In addition, PSMA expression correlates with recurrence and progression of prostate cancer, as well as to the presence of metastases [5,6]. Due to lack of physiological expression in normal tissue of common metastatic sites of prostate cancer, and its advantages compared to conventional imaging, PSMA positron emission tomography (PET) imaging has become a major diagnostic tool in mCRPC patients [7,8,9] After the great success of PSMA based imaging, the application of PSMA targeted therapies with alpha- or beta-emitters was only a matter of time. After the report of the VISION-data and PSMA RLT on its way to approval, this review briefly summarizes the application, eligibility and the current state of safety and efficacy of ^177^Lutetium (Lu)-PSMA therapy.

## 2. Eligibility and Procedure Guidelines

To this point, ^177^Lu-PSMA RLT can only be administered as an experimental treatment approach. Before considering this treatment, the currently approved therapeutical options need to be applied in advance. Thus, most of these patients, next to conventional androgen deprivation therapy (ADT), already received new antihormonal therapy using enzalutamide and/or abiraterone and, if possible, first- and/or second-line chemotherapy with docetaxel and cabazitaxel [10,11,12,13]. Another therapy only approved by the FDA is Sipuleucel-T. This is, however, mostly unknown in European countries [14]. In addition, the Poly(ADP-Ribose)-Polymerase 1 (PARP)-inhibitor olaparib and rucaparib, novel therapies, can be offered to patients with mCRPC if an inactivated Breast Cancer ½ gene is detectable [15,16].

As ^177^Lu-PSMA RLT is still lacking approval. there are no official procedure guidelines as to when and how to use this treatment approach. However, the European Association of Nuclear Medicine (EANM) released a non-binding procedure guideline, and a group of practitioners around Ahmadzadehfar et al. published a step-by-step practical approach to help and assist those applying ^177^Lu-PSMA RLT [17,18].

Before considering a therapy with ^177^Lu-PSMA, patients should be examined and have an Eastern Cooperative Oncology Group performance status (ECOG) ≤ 2. The total white blood cell count should be higher than 2–2.5 × 10^9^/L, and the platelet count should at least be over 75 × 10^9^/L. It is stated that a sufficient kidney function is also important for the eligibility for therapy. A creatinine two-fold upper limit of normal or a glomerular filtration rate lower than 30–40 mL/min/1.73 m^2^ are said to be a contraindication for therapy. To assess the renal function, and to rule out any urinary obstruction, a ^99m^Technetium (Tc)-Mercaptoacetyltriglycerine (MAG3) or ^99m^Tc-diethylenetriaminepentaacetic acid (DTPA) renal scintigraphy can be performed as a baseline examination. Any urinary tract obstruction should be dissolved prior to therapy to reduce the risk of possible side effects. This recommendation, however, is based on limited scientific evidence [17,18]. If these above-mentioned criteria are met, the medical indication for a ^177^Lu-PSMA therapy should be discussed by an interdisciplinary tumour board. Each patient must undergo PSMA imaging, for example ^68^Gallium (Ga)-PSMA-PET, to prove the presence and to quantify the amount of uptake of PSMA in tumour lesions. Whether the amount of PSMA-tracer uptake has an impact on the following RLT and its efficacy remains unclear to this point [19,20,21,22,23].

The amount of ^177^Lu-PSMA activity administered per cycle ranges between about 4 and 9 GBq in previous studies [24,25,26,27]. Relying on this data, the EANM states that an activity between 3.7–9.3 GBq per cycle can be applied and the time between cycles should be 6–8 weeks [17]. The prospective trials published so far administered ^177^Lu-PSMA in 6-week intervals with an activity of 6–8.5 GBq [21,28,29,30,31] However, in a retrospective analysis from Rasul et al., cycles in a 4-week interval with an administered activity of 7.4 GBq seemed to offer favourable outcomes regarding median progression-free survival, survival rates, and response rates without a significant increase in adverse events [32].

Immediately before starting the therapy, patients should be hydrated sufficiently. About 500–1000 mL intravenous Ringer or 0.9% Natrium-Chloride solution is recommended [17]. Patients suffering from nausea and vomiting can receive an antiemetic drug, for example any 5-hydroxytryptamine 3 receptor antagonist. To reduce the risk of posttreatment xerostomia, cooling of the salivary gland is still under discussion [17,18].

A thorough follow-up regime is necessary for patients receiving ^177^Lu-PSMA RLT. Potential side effects, as well as the response to therapy, need to be monitored and evaluated. According to the step-by-step practical approach and the EANM procedure guidelines, blood tests (complete blood count, liver, and kidney profile), as well as clinical examinations, should be performed every 2–8 weeks [17,18]. In 4-week intervals, prostate-specific antigen (PSA) tests should be performed to assess the biochemical response under RLT with ^177^Lu-PSMA [18]. Imaging, favourably PSMA imaging, is recommended after two to four cycles [17,18]. Ahmadzadehfar et al. also suggest that patients without any PSA response after 2 cycles should have an earlier restaging performed [18].

## 3. Efficacy and Safety

Although patients treated with ^177^Lu-PSMA RLT are mostly at an advanced stage of mCRPC, and already have had many different previous systemic therapies, there are to date promising results published according to efficacy and safety of this new therapeutic. In these studies, the patients showed a good response to therapy, whilst suffering only from limited and moderate adverse events.

### 3.1. Prostate-Specific Antigen Response

The first retrospective study using Lu-PSMA RLT was published in 2015 [33]. Ahmadzadehfar et al. described the early side effects and the PSA response of RLT with ^177^Lu-DKFZ-617 PSMA in 10 patients with progressive end-stage mCRPC. All patients received only 1 cycle of therapy and after 2 months the response was evaluated. A total of 7 out of these 10 patients experienced a PSA decline, and in five of these patients the PSA decline was greater than 50%. After receiving two cycles of ^177^Lu-DKFZ-617 PSMA RLT the PSA response seemed to be even better [34]. Two months later, 60% of the 22 patients enrolled in that study had a PSA decline over 50%. In another study by Ahmadzadehfar et al. it was shown that about 50% of the patients, with no PSA response after the first cycle, responded after the second or third cycle [35].

The German multicentre study was the largest cohort of patients with advanced prostate cancer receiving PSMA RLT published in 2017 [24]. In this retrospective study, 145 patients with mCRPC received at least one, and up to four, cycles of ^177^Lu-PSMA-617. In 99 patients, the biochemical response was analysed. Forty-five percent of these patients were considered biochemical responders, with a PSA decline of over 50%. Any PSA decline was detected in 60% of the patients. After one cycle, a PSA decline over 50% occurred in 40 of 99 patients (40%). After the second, third, and fourth cycle a PSA decline over 50% was detected in 35 of 61 (57%), 13 of 20 (65%), and 3 of 3 patients (100%), respectively. In this cohort, visceral metastases, as well as elevated alkaline phosphate, were associated with a less significant PSA response. In another more recently published multicentre study, similar results can be seen [36]. Data from 631 patients were collected and the PSA values of 393 patients were available two months after the first cycle. In these patients, a PSA decline over 50% occurred in 42%, and any PSA decline in 72%. A PSA increase, however, occurred in 28% patients after receiving only one cycle of ^177^Lu-PSMA RLT. The PSA response of the above-mentioned publications is summarized in Table 1.

A systemic review and meta-analysis including 36 original research publications with in total 2346 mCRPC patients receiving PSMA therapy has been published by von Eyben et al. in 2020 [49]. After the first and second cycle, half of these patients had a PSA decline over 50%. Patients with a PSA response over 50% had a prolonged survival compared to those with less or no decline (20 months vs. 12 months). However, patients with PSA progression, and those with a PSA decline of less than 50%, had a similar median overall survival.

**Figure 1:**
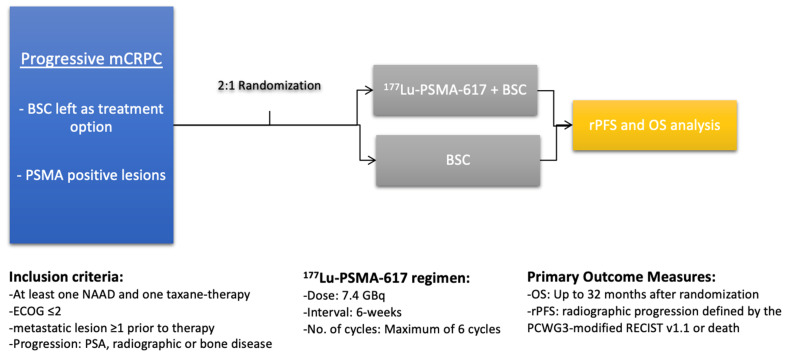
Outline of the Vision phase III trial. Abbreviations: BSC: Best supportive care, rPFS: radiographic Progression-free survival, OS: overall survival, NAAD: novel androgen axis drug, and PCWG3: Prostate Cancer Working Group 3.

The first prospective study on 177Lu-PSMA therapy is an Australian single-centre, single-arm phase 2-study by Hofman et al. [21]. In this study, 43 patients with mCRPC were included. All patients underwent imaging with ^68^Ga-PSMA-11, as well as with ^18^F-fluorodeoxyglucose (FDG) PET/computed tomography (CT). PSMA PET imaging was performed to evaluate metastatic sites with high PSMA expression (defined as standardised uptake value (SUV)_max_) of tumour involvement at least 1.5× SUV of the liver). These patients, and patients with low PSMA expression in general, were excluded and did not receive ^177^Lu-PSMA RLT. The remaining 30 patients where eligible for therapy. A PSA decline of at least 50% or higher was achieved in 17 patients (57%). Any PSA decline was reported in 29 cases (97%). This number is higher than in previous retrospective studies. A reason for this finding is likely that only patients with a high PSMA expression were included, which probably results in higher ^177^Lu-PSMA uptake in tumour lesions. Moreover, patients with FDG-positive metastases and low or failing PSMA uptake in metastatic lesions were excluded. FDG-uptake is associated with tumour aggressiveness [50]. This prospective trial was prolonged and expanded up to 50 treated patients [48]. A PSA response ≥50% was noted in even more patients in this expanded cohort (64% vs. 57%). A recently published phase II trial (TheraP trial), also by Hofman et al., compared in a randomised cohort of 200 mCRPC patients Lu-PSMA therapy to a treatment with cabazitaxel [29]. A total of 98 patients received up to 6 cycles ^177^Lu-PSMA-617, whereas 85 were treated with up to 10 cycles of cabazitaxel. The primary endpoint was a reduction of ≥50% of the initial PSA value. A total of 66% of the patients treated with ^177^Lu-PSMA-617 had a PSA reduction over 50%, whereas in the cabazitaxel control group this was only achieved by 44%. This was the first study comparing PSMA therapy to another, already approved therapeutic. Thus, these prospective trials have the potential to change treatment regimens of progressing mCRPC patients. Table 1 gives an overview to compare these prospective results with previous published retrospective data. In the recently presented results of the VISION phase III trial, patients who already received at least one androgen receptor pathway inhibitor and one taxane chemotherapy were randomised 2:1, either receiving ^177^Lu-PSMA-617 RLT plus standard of care, or protocol-permitted standard of care alone [31]. The VISION trial study outline is illustrated in Figure 1 (adapted after Rahbar et al. [28]). A total of 551 patients received PSMA therapy, and a PSA reduction of over 50% was reported in 46% of the cases, compared to 7.1% in the control group. 

**Figure 2:**
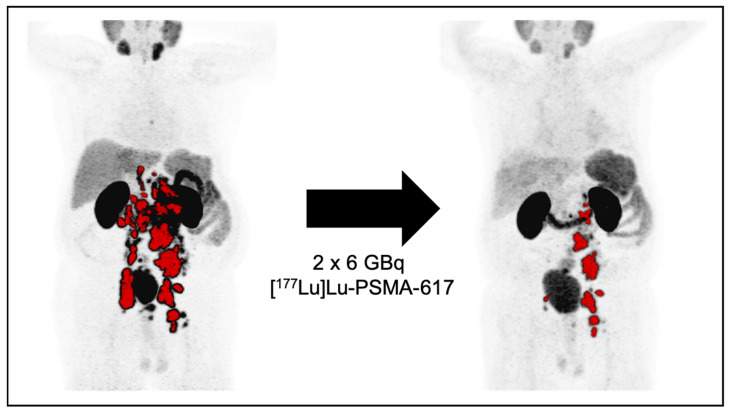
Patient case after two cycles with ^177^Lu-PSMA RLT. Patient with mCRPC, previously treated with Docetaxel, Abiraterone, and Enzalutamide. Pre Lu-PSMA therapy PSA levels were 791 ng/mL, after two cycles of Lu-PSMA therapy, PSA levels declined to 114 ng/mL. Concordantly, the PSMA PET derived tumor volume declined from 245 mL to 55 mL.

Many, mainly retrospective, studies have shown a significant PSA response after ^177^Lu-PSMA RLT, especially after administering repeated cycles. In addition, any PSA decline, as well as a PSA decline greater than 50%, seemed to be a significant prognosticator of prolonged overall survival [21,37,38,39,48,51]. The few prospective trials published so far were able to confirm these retrospective findings [21,29,48] A PSA decline may also correlate with a PSMA PET tumour volume reduction. This can be demonstrated in the following case. A PSA decline far over 50% after two cycles of RLT significantly correlated with a reduction in the tumour volume. Figure 2 shows these impressive findings. 

### 3.2. Progression-Free and Overall Survival 

At this moment, except the VISION trial, mostly retrospective and single-arm prospective studies evaluating overall survival have been published. These results can only be compared to historical survival data, making it difficult to accurately predict the effects of ^177^Lu-PSMA RLT.

In 2016, Rahbar et al. were the first to estimate median overall survival [40]. In this investigation, 28 patients with mCRPC were treated with up to two cycles of ^177^Lu-PSMA-617. The overall survival was reported to be 29.4 weeks, which was longer compared with best supportive care of previous historic data of patients with matched clinical data, prior to the availability of ^177^Lu-PSMA-617. Two years later, a study published by the same group of investigators, which included 104 patients with mCRPC receiving, in total, 351 cycles (with a median of three cycles) of ^177^Lu-PSMA-617, showed an even longer median overall survival of 56 weeks [41]. After these first promising results, further studies have been published to support these initial findings. The largest cohort so far included 631 patients with end-stage prostate cancer [36]. Patients had a median overall survival of 11.1 months with a median of three applied cycles. In the already mentioned meta-analysis, the calculated median overall survival after ^77^Lu-PSMA RLT was said to be 16 months. To this point, overall survival was reached in only one prospective patient cohort [21,48]. Here, the median overall survival was 13.3 months, the median PSA progression-free survival of these 50 patients, of whom all had a PSA progression during observation, was 6.9 months. 

The median progression-free survival in the prospective trial by Hofman et al. was 5.1 months [29] albeit not being significantly different between the RLT and cabazitaxel chemotherapy group. However, after 12 months, the median progression-free survival in the RLT group was 19% and in the cabazitaxel group 3%. This difference was statistically significant.

The previously mentioned VISION phase III trial, which included 831 patients with progressive mCRPC, analysed overall survival and radiographic progression-free survival (rPFS) after ^177^Lu-PSMA-617 RLT in comparison with standard/best supportive care [31]. The overall survival in the RLT group was 15.3 months vs. 11.3 months in the control group. The difference was statistically significant. The rPFS was also significantly longer in the treatment group (8.7 vs. 3.4 months). These newly published results verify the efficacy of ^177^Lu-PSMA-617 RLT. An overview of the survival analyses in different trials can be found in Table 1.

A change in the treatment regime might prolong overall survival. One group of researchers shortened the interval between each cycle of ^177^Lu-PSMA therapy [32]; 54 patients received three cycles every 4 weeks. In this retrospective analysis, the median overall survival was 119 weeks, which is longer compared to previous retrospective data with treatment cycles every six to eight weeks. However, there are limitations comparing these studies and data on overall survival or progression-free survival, due to the retrospective manner, as well as different inclusion and exclusion criteria. A prospective trial is required to evaluate whether or not a shorter treatment interval results in favourable outcome. 

Many different factors have been identified to be prognosticators for overall survival of ^177^Lu-PSMA RLT. We already know from other therapies for advanced prostate cancer that the presence of visceral metastases, especially liver metastases, and an ECOG ≥2 are negative prognosticators for overall survival as well as progression-free survival. Retrospective data published to date shows that this might also be true for RLT [36,38,39,42]. Two independent groups of investigators reported that patients with presence of only lymph node metastases had the longest median overall survival [33,43]. Interestingly, prior taxane-chemotherapy, irrespective of receiving first- or second-line chemotherapy, was also associated with a shorter median overall survival [36,38,44]. However, prior abiraterone, enzalutamide, or Radium-223 treatment seemed to have no influence on prognosticating median overall survival [36]. As already mentioned, a PSA-Response ≥50% prognosticates a prolonged median overall survival. Other laboratory findings, such as haemoglobin, lactate dehydrogenase, alkaline phosphatase, and different liver enzymes, are still under discussion as to whether or not they have a prognostic value [37,39,45,46,47].

### 3.3. Imaging and Response

There is still a great number of patients who do not respond to ^177^Lu-PSMA therapy. We previously discussed some of the predicting and prognosticating factors. However, it is important to know and understand which patients benefit the most from RLT, and how treatment regimens could be adapted in the future based on imaging findings. In recently published studies, results and parameters of pretherapeutic imaging have gained a lot of interest [21,22,48,52,53,54,55,56]. In these publications, either the total tumour volume or the level of PSMA expression was quantitatively assessed in pretherapeutic PET-imaging to evaluate the predictive and prognostic value. Regarding the tumour volume, its prognostic value is still under discussion. Ferdinandus et al. stated that the tumour volume determined in PSMA PET-imaging does not have any prognostic value, whereas the one determined in FDG-PET does [54]. Contrary to these results, Seifert et al. found that, in two different patient cohorts, the determined tumour volume in PSMA PET-imaging was a strong prognosticator [52,53]. Seifert et al. observed in a retrospective analysis of 110 patients in two centres in Germany a correlation of tumour volume and survival outcome [53]. Although the tumour volume did not remain statistically significant in the multivariate analysis, another significant factor was identified. The total lesion quotient (tumour volume * SUV_mean_) was an independent prognosticator of median overall survival, even in the multivariate Cox regression analyses.

PSMA expression might predict and prognosticate response and outcome [19,20,22,23]. It seems logical that a higher PSMA expression is linked to a better response to PSMA targeted RLT. This is why the EANM guidelines recommend a PSMA PET-imaging prior to therapy. Although it is important to understand and know which mCRPC patients are mostly suited for ^177^Lu-PSMA RLT, it is unclear, due to lack of publications, to what extent the PSMA expression has an impact on response and outcome. In the prospective phase II trial of Hofman et al., they defined high PSMA expression with the use of a liver specific threshold (1.5 * SUV_mean_ liver) [21,48]. Patients that did not reach this threshold were ineligible for RLT with ^177^Lu-PSMA [56]. This, however, was a pragmatical approach without any scientific evidence. Seifert et al. tried to identify ^68^Ga-PSMA PET-CT parameters with prognostic value [22]. In these 85 patients, they identified that the average SUV_max_ of all metastases and the SUV_max_ of the lowest PSMA expressing metastasis have a prognostic value. A cut-off was calculated for these two parameters to compare survival of high and low PSMA expressing groups. In the groups with low PSMA expression in their metastases, the median overall survival was significantly shorter, and thus low PSMA expressions seems to be a negative prognosticator for mCRPC patients treated with ^177^Lu-PSMA. 

### 3.4. Safety

^177^Lu-PSMA has shown only limited-to-none severe and mainly moderate adverse events in studies published to date [21,24,27,30,32,39,44,48] (Table 2). All results discussed below were categorized using the Common Terminology Criteria for Adverse Events (CTCAE) version 4.03. A large retrospective multicentre study analysed the toxicity of ^177^Lu-PSMA RLT in 145 mCRPC patients [24]. Rahbar et al. observed Grade 3–4 haematologic adverse events in 12% of the patients. Most of them (10%) suffered from anaemia. Other Grade 3–4 adverse events only occurred in individual cases. Besides the most commonly occurring haematologic adverse events (40% with leukopenia, 34% with anaemia, and 31% with thrombocytopenia), an elevation of aspartate aminotransferase (AST) and alanine aminotransferase (ALT) was observed in 19% and 8%, respectively. Patients reported to suffer from xerostomia in 8%, nausea in 6%, and fatigue in 13% of the cases. Nephrotoxicity Grade 1–2 occurred in 12% and Grade 3–4 in no patients. Due to its retrospective nature, a common limitation might be underreporting and -documentation of adverse events. Therefore, prospective trials are better suited to monitor adverse events. Observed adverse events in the prospective trials were in line with previously discussed results on haematological toxicity of the German multicentre trial [21,29,31,48]. Only Grade 3 and 4 thrombocytopenia was detected in more cases (11% vs. 4%) Leukopenia was even seen in fewer patients receiving ^177^Lu-PSMA RLT. In another prospective trial of the same group, and the Vision trial, the described side effects were fairly similar [21,31,48]. One notable difference, however, was that Grade 3–4 lymphocytopenia was detected in 43% and 32%. Unfortunately, a statement about the white blood cell count is missing. 

Regarding other side effects, the prospective TheraP trial by Hofman et al. reported pain (all Grades 72%), fatigue (all Grades 75%), nausea (all Grades 41%), and xerostomia (all Grades 60%) more often compared to previously published retrospective reports [29]. The huge difference reporting xerostomia and nausea can be related to different study protocols only partially including cooling of the salivary glands and giving antiemetic drugs in advance. In comparison to cabazitaxel, ^177^Lu-PSMA showed significantly fewer severe adverse events in this trial. Treating patients with a higher activity of ^177^Lu-PSMA, with a shorter interval between each cycle, did not cause significantly more adverse events in two different retrospective analyses [30,32].

## 4. Future Perspectives

Seeing the results of many retrospective and a few prospective trials, we can expect that ^177^Lu-PSMA RLT is going to be an important treatment option for patients with advanced prostate cancer. The trial by Hofman et al. comparing cabazitaxel, as a standard of treatment, to ^177^Lu-PSMA-617 has given the first promising results regarding the response [29]. The survival analysis and the final results of the progression-free survival are yet to come, and are eagerly awaited within the next year. Retrospective data has shown that patients with prior taxane-chemotherapy had worse survival outcome after RLT. Taking this into account, and the fact that, according to the TheraP trial, ^177^Lu-PSMA might be applied prior to cabazitaxel in the treatment of mCRPC, it is necessary to discuss if ^177^Lu-PSMA RLT might be used in earlier stages in patients with advanced prostate cancer, maybe even prior or in addition to hormone therapy. Currently, there are no prospective trials evaluating the right sequencing of treatment options, as well as head-to-head studies comparing the available therapies in mCRPC. Therefore, it is even more challenging to choose the best treatment for each individual patient at the ideal time. Nonetheless, some trials are already on the way to evaluate that matter (e.g., LuPARP (NCT03874884), Enza-p (NCT04419402) or SPLASH (NCT04647526)).

Many questions regarding eligibility, safety and efficacy of ^177^Lu-PSMA RLT are still under discussion. The procedure guidelines by the EANM still include many recommendations needing prospective scientific validation [17]. It is also uncertain to what extent PSA reduction is reliable as a marker for response evaluation. In a recently published systemic review and meta-analysis by Han et al., a discordance between PSMA PET and PSA response was described in nearly a fourth of mCRPC patients [57]. Reasons for this might be of heterogenous origin. Regarding RLT, this described phenomena should be investigated further in large prospective trials to better understand the efficacy of ^177^Lu-PSMA-617. Safety measurements, and especially prevention of adverse events, still need to be discussed, and future studies should work on how to further reduce side effects of ^177^Lu-PSMA RLT. Two phase II studies will bring more light to safety issues during and after therapy (RESIST-PC [58] (NCT03042312) and LU-PSMA (NCT03454750)). 

The eligibility criteria based on PSMA PET prior to therapy will be another point of discussion in future studies, and need to be prospectively validated. 

Recently, alpha-emitters, namely ^225^Actinium (Ac)-PSMA, were applied in mCRPC patients. Quite a number of retrospective data has already been published, and a prospective trial is already recruiting participants (NCT04597411) [59,60,61,62,63]. In addition, a tandem therapy of ^177^Lu- and ^225^Ac-PSMA had shown benefits in reducing adverse events of ^225^Ac-PSMA and in improving response [64]. 

The benefit of RLT with ^177^Lu-PSMA in mCRPC patients has already been demonstrated in many publications. Thus, many researchers now focus on investigating its impact in earlier stages of the disease. As a neoadjuvant treatment to improve outcome for high-risk localized prostate cancer prior to prostatectomy, two prospective trials are currently recruiting (LuTectomy (NCT04430192) and NALuPROST (NCT04297410)). The same working groups also initiated the investigation of application in a metastatic hormone sensitive stage (UpFrontPSMA (NCT 04343885) and two trials by the Radboud University (NCT03828838 and NCT04443062)).

## 5. Conclusions

With the approval of olaparib and rucaparib last year, there is already a tremendous amount of approved treatment options in mCRPC. Large prospective trials, and the recently published TheraP and phase III VISION trial will pave the way for approval of ^177^Lu-PSMA therapy within this year. This makes it even more challenging for clinicians to choose the ideal treatment for mCRPC patients. More prospective trials are needed to define the role of PSMA therapy in the treatment regimens in mCRPC patients. Thus, in the future, ^177^Lu-PSMA might also play a role in earlier stages of prostate cancer. Moreover, interdisciplinary tumour boards are indispensable for planning treatment of patients with advanced prostate cancer. 

## Figures and Tables

**Table 1:** Overview of treatment characterisitics, PSA response ≥ 50% and overall survival of ^177^Lu-PSMA RLT in published retrospective and prospective trails.

Reference	n	Dose per Cycle(GBq)	Median No of Cycles	Interval between Cycles (Weeks)	PSA Decline ≥ 50%(%)	Overall Survival (Months)
**Retrospective trials**						
Rahbar et al. [24]	145	2.0–8.0	2	8–12	45	Not reported
Rasul et al. [32]	54	7.3	3	4	54	28
Ahmadzadehfar et al. [34]	22	4.1–7.1	2	Not reported	60	Not reported
Ahmadzadehfar et al. [35]	52	4.0–7.2	1	/	44	13.8
Ahmadzadehfar et al. [36]	393	6.9	1	/	42	11.1
Braeuer et al. [37]	59	6.1	3	8	53	8
Kessel et al. [38]	109	6.2	3	6–8	25	9.9
Heck et al. [39]	100	7.4	2	6–8	38	12.9
Rahbar et al. [40]	28	6.0	2	8	32	6.8
Rahbar et al. [41]	104	6.1	3	8	33	14
Khreish et al. [42]	28	6.5	4	6	57	12
von Eyben et al. ^1^ [43]	45	4.6	3	8	80	42
Barber et al. [44]	167	6.3	3	6	48	18
Ahmadzadehfar et al. [45]	100	6.0	3	8	38	15
Rathke et al. [46]	100	Not reported	2	8	35	Nor reported
Ferdinandus et al. [47]	40	6.1	1	/	32.5	Not reported
**Prospective trails**						
Hofman et al. [21]	30	4.4–8.7	1–4	6	57	13.5
Violet et al. [48]	50	4.0–8.5	4	6	64	13.3
Hofman et al. [29]	98	6.0–8.5	5	6	66	Not reported
-Vs. Cabazitaxel	85	/	/	/	37	Not reported
Sartor et al. [31]	551	7.4	6	6	46	15.3
-Vs. Standard of care	280		/	/		11.3

^1^ only patients with lymphnode metastases were included.

**Table 2:** Adverse events, classified by CTCAE, after ^177^Lu-PSMA RLT observed in different publications.

Reference	n	Haematotoxicity-Grade 3 & 4 (%)			Other AE-All Grades (%)			
		Hb	WBC	Plt	Renal Injury	Dry Mouth	Nausea	Fatigue
**Retrospective trails**								
Rahbar et al. [24]	145	10	3	4	12	8	6	13
Baum et al. [27]	56	0	0	0	0	3.5	NR	NR
Seifert et al. [30]	78	23	5	5	10	15	10	NR
Rasul et al. [32]	54	6	6	0	0	NR	NR	NR
Heck et al. [39]	100	9	6 ^1^	4	/	24	/	20
Barber et al. [44]	167	5	1	2	25	NR	NR	NR
**Prospective trails**								
Hofman et al. [21]	30	13	37 ^2^	13	NR	87	50	50
Violet et al. [48]	50	10	32 ^2^	10	NR	66	48	38
Hofman et al. [29]	98	8	1	11	NR	60	41	75
Sartor et al. [31]	529	13	9	8	9	39	39	49

^1^ only Neutropenia reported, ^2^ only Lymphocytopenia reported. Abbreviations: Hb: haemoglobin, WBC: white blood cells, Plt: platlets, AE: adverse events, and NR: not reported.

## References

[1] Mhawech-Fauceglia P., Zhang S., Terracciano L., Sauter G., Chadhuri A., Herrmann F.R., Penetrante R. (2007). Prostate-Specific Membrane Antigen (PSMA) Protein Expression in Normal and Neoplastic Tissues and Its Sensitivity and Specificity in Prostate Adenocarcinoma: An Immunohistochemical Study Using Mutiple Tumour Tissue Microarray Technique. Histopathology.

[2] Silver D.A., Pellicer I., Fair W.R., Heston W.D.W., Cordon-Cardo C. (1997). Prostate-Specific Membrane Antigen Expression in Normal and Malignant Human Tissues. Clin. Cancer Res..

[3] Wang X., Yin L., Rao P., Stein R., Harsch K.M., Lee Z., Heston W.D.W. (2007). Targeted Treatment of Prostate Cancer. J. Cell. Biochem..

[4] Sheikhbahaei S., Afshar-Oromieh A., Eiber M., Solnes L.B., Javadi M.S., Ross A.E., Pienta K.J., Allaf M.E., Haberkorn U., Pomper M.G. (2017). Pearls and Pitfalls in Clinical Interpretation of Prostate-Specific Membrane Antigen (PSMA)-Targeted PET Imaging. Eur. J. Nucl. Med. Mol. Imaging.

[5] Perner S., Hofer M.D., Kim R., Shah R.B., Li H., Möller P., Hautmann R.E., Gschwend J.E., Kuefer R., Rubin M.A. (2007). Prostate-Specific Membrane Antigen Expression as a Predictor of Prostate Cancer Progression. Hum. Pathol..

[6] Minner S., Wittmer C., Graefen M., Salomon G., Steuber T., Haese A., Huland H., Bokemeyer C., Yekebas E., Dierlamm J. (2011). High Level PSMA Expression Is Associated with Early Psa Recurrence in Surgically Treated Prostate Cancer. Prostate.

[7] Afshar-Oromieh A., Holland-Letz T., Giesel F.L., Kratochwil C., Mier W., Haufe S., Debus N., Eder M., Eisenhut M., Schäfer M. (2017). Diagnostic Performance Of68Ga-PSMA-11 (HBED-CC) PET/CT in Patients with Recurrent Prostate Cancer: Evaluation in 1007 Patients. Eur. J. Nucl. Med. Mol. Imaging.

[8] Schwarzenboeck S.M., Rauscher I., Bluemel C., Fendler W.P., Rowe S.P., Pomper M.G., Afshar-Oromieh A., Herrmann K., Eiber M. (2017). PSMA Ligands for PET Imaging of Prostate Cancer. J. Nucl. Med..

[9] Weber M., Hadaschik B., Ferdinandus J., Rahbar K., Bögemann M., Herrmann K., Fendler W.P., Kesch C. (2021). Prostate-Specific Membrane Antigen-Based Imaging of Castration-Resistant Prostate Cancer. Eur. Urol. Focus.

[10] Beer T.M., Armstrong A.J., Rathkopf D.E., Loriot Y., Sternberg C.N., Higano C.S., Iversen P., Bhattacharya S., Carles J., Chowdhury S. (2014). Enzalutamide in Metastatic Prostate Cancer before Chemotherapy. N. Engl. J. Med..

[11] Ryan C.J., Smith M.R., Fizazi K., Saad F., Mulders P.F.A., Sternberg C.N., Miller K., Logothetis C.J., Shore N.D., Small E.J. (2015). Abiraterone Acetate plus Prednisone versus Placebo plus Prednisone in Chemotherapy-Naive Men with Metastatic Castration-Resistant Prostate Cancer (COU-AA-302): Final Overall Survival Analysis of a Randomised, Double-Blind, Placebo-Controlled Phase 3 Study. Lancet Oncol..

[12] Berthold D.R., Pond G.R., Soban F., De Wit R., Eisenberger M., Tannock I.F. (2008). Docetaxel plus Prednisone or Mitoxantrone plus Prednisone for Advanced Prostate Cancer: Updated Survival in the TAX 327 Study. J. Clin. Oncol..

[13] De Bono J.S., Oudard S., Ozguroglu M., Hansen S., MacHiels J.P., Kocak I., Gravis G., Bodrogi I., MacKenzie M.J., Shen L. (2010). Prednisone plus Cabazitaxel or Mitoxantrone for Metastatic Castration-Resistant Prostate Cancer Progressing after Docetaxel Treatment: A Randomised Open-Label Trial. Lancet.

[14] Penson D.F., Redfern C.H., Ferrari A.C., Dreicer R., Sims R.B., Xu Y., Ph D., Frohlich M.W., Schellhammer P.F. (2010). Sipuleucel-T Immunotherapy for Castration-Resistant Prostate Cancer. N. Engl. J. Med..

[15] De Bono J., Mateo J., Fizazi K., Saad F., Shore N., Sandhu S., Chi K.N., Sartor O., Agarwal N., Olmos D. (2020). Olaparib for Metastatic Castration-Resistant Prostate Cancer. N. Engl. J. Med..

[16] Abida W., Patnaik A., Campbell D., Shapiro J., Bryce A.H., McDermott R., Sautois B., Vogelzang N.J., Bambury R.M., Voog E. (2020). Rucaparib in Men with Metastatic Castration-Resistant Prostate Cancer Harboring a BRCA1 or BRCA2 Gene Alteration. J. Clin. Oncol..

[17] Kratochwil C., Fendler W.P., Eiber M., Baum R., Bozkurt M.F., Czernin J., Bolton R.C.D., Ezziddin S., Forrer F., Hicks R.J. (2019). EANM Procedure Guidelines for Radionuclide Therapy with 177 Lu-Labelled PSMA-Ligands ( 177lu-PSMA-RLT ). Eur. J. Nucl. Med. Mol. Imaging.

[18] Ahmadzadehfar H., Rahbar K., Essler M., Biersack H.J. (2020). PSMA-Based Theranostics: A Step-by-Step Practical Approach to Diagnosis and Therapy for MCRPC Patients. Semin. Nucl. Med..

[19] Khurshid Z., Ahmadzadehfar H., Gaertner F.C., Papp L., Zsóter N., Essler M., Bundschuh R.A. (2018). Role of Textural Heterogeneity Parameters in Patient Selection for 177Lu-PSMA Therapy via Response Prediction. Oncotarget.

[20] Emmett L., Crumbaker M., Ho B., Willowson K., Eu P., Ratnayake L., Epstein R., Blanksby A., Horvath L., Guminski A. (2019). Results of a Prospective Phase 2 Pilot Trial of 177 Lu–PSMA-617 Therapy for Metastatic Castration-Resistant Prostate Cancer Including Imaging Predictors of Treatment Response and Patterns of Progression. Clin. Genitourin. Cancer.

[21] Hofman M.S., Violet J., Hicks R.J., Ferdinandus J., Ping Thang S., Akhurst T., Iravani A., Kong G., Ravi Kumar A., Murphy D.G. (2018). [ 177 Lu]-PSMA-617 Radionuclide Treatment in Patients with Metastatic Castration-Resistant Prostate Cancer (LuPSMA Trial): A Single-Centre, Single-Arm, Phase 2 Study. Lancet Oncol..

[22] Seifert R., Seitzer K., Herrmann K., Kessel K., Schaefers M., Kleesiek J., Weckesser M., Boegemann M., Rahbar K. (2020). Analysis of PSMA Expression and Outcome in Patients with Advanced Prostate Cancer Receiving 177 Lu-PSMA-617 Radioligand Therapy. Theranostics.

[23] Vlachostergios P.J., Niaz M.J., Skafida M., Mosallaie S.A., Thomas C., Christos P.J., Osborne J.R., Molina A.M., Nanus D.M., Bander N.H. (2021). Imaging Expression of Prostate-Specific Membrane Antigen and Response to PSMA-Targeted β-Emitting Radionuclide Therapies in Metastatic Castration-Resistant Prostate Cancer. Prostate.

[24] Rahbar K., Ahmadzadehfar H., Kratochwil C., Haberkorn U., Schafers M., Essler M., Baum R.P., Kulkarni H.R., Schmidt M., Drzezga A. (2017). German Multicenter Study Investigating 177 Lu-PSMA-617 Radioligand Therapy in Advanced Prostate Cancer Patients. J. Nucl. Med..

[25] Heck M.M., Retz M., D’Alessandria C., Rauscher I., Scheidhauer K., Maurer T., Storz E., Janssen F., Schottelius M., Wester H.J. (2016). Systemic Radioligand Therapy with 177Lu Labeled Prostate Specific Membrane Antigen Ligand for Imaging and Therapy in Patients with Metastatic Castration Resistant Prostate Cancer. J. Urol..

[26] Rathke H., Giesel F.L., Flechsig P., Kopka K., Mier W., Hohenfellner M., Haberkorn U., Kratochwil C. (2018). Repeated 177 Lu-Labeled PSMA-617 Radioligand Therapy Using Treatment Activities of up to 9.3 GBq. J. Nucl. Med..

[27] Baum R.P., Kulkarni H.R., Schuchardt C., Singh A., Wirtz M., Wiessalla S., Schottelius M., Mueller D., Klette I., Wester H.J. (2016). 177Lu-Labeled Prostate-Specific Membrane Antigen Radioligand Therapy of Metastatic Castration-Resistant Prostate Cancer: Safety and Efficacy. J. Nucl. Med..

[28] Rahbar K., Bodei L., Morris M.J. (2019). Is the Vision of Radioligand Therapy for Prostate Cancer Becoming a Reality? An Overview of the Phase III VISION Trial and Its Importance for the Future of Theranostics. J. Nucl. Med..

[29] Hofman M.S., Emmett L., Sandhu S., Iravani A., Joshua A.M., Goh J.C., Pattison D.A., Tan T.H., Kirkwood I.D., Ng S. (2021). [177Lu]Lu-PSMA-617 versus Cabazitaxel in Patients with Metastatic Castration-Resistant Prostate Cancer (TheraP): A Randomised, Open-Label, Phase 2 Trial. Lancet.

[30] Seifert R., Kessel K., Schlack K., Weckesser M., Boegemann M., Rahbar K. (2020). Radioligand Therapy Using [177Lu]Lu-PSMA-617 in MCRPC: A Pre-VISION Single-Center Analysis. Eur. J. Nucl. Med. Mol. Imaging.

[31] Sartor O., de Bono J., Chi K.N., Fizazi K., Herrmann K., Rahbar K., Tagawa S.T., Nordquist L.T., Vaishampayan N., El-Haddad G. (2021). Lutetium-177-PSMA-617 for Metastatic Castration-Resistant Prostate Cancer. N. Engl. J. Med..

[32] Rasul S., Hacker M., Kretschmer-Chott E., Leisser A., Grubmüller B., Kramer G., Shariat S., Wadsak W., Mitterhauser M., Hartenbach M. (2020). Clinical Outcome of Standardized 177Lu-PSMA-617 Therapy in Metastatic Prostate Cancer Patients Receiving 7400 MBq Every 4 Weeks. Eur. J. Nucl. Med. Mol. Imaging.

[33] Ahmadzadehfar H., Rahbar K., Kuerpig S., Boegemann M., Claesener M., Eppard E., Gaertner F., Rogenhofer S., Schaefers M., Essler M. (2015). Early Side Effects and First Results of Radioligand Therapy with 177Lu-DKFZ-617 PSMA of Castrate-Resistant Metastatic Prostate Cancer: A Two-Centre Study. EJNMMI Res..

[34] Ahmadzadehfar H., Eppard E., Kuerpig S., Fimmers R., Yordanova A., Schlenkhoff C.D., Gaertner F., Rogenhofer S., Essler M. (2016). Therapeutic Response and Side Effects of Repeated Radioligand Therapy with 177Lu-PSMA-DKFZ-617 of Castrate-Resistant Metastatic Prostate Cancer. Oncotarget.

[35] Ahmadzadehfar H., Wegen S., Yordanova A., Fimmers R., Kuerpig S., Eppard E., Wei X., Schlenkhoff C., Hauser S., Essler M. (2017). Overall Survival and Response Pattern of Castration-Resistant Metastatic Prostate Cancer to Multiple Cycles of Radioligand Therapy Using [177Lu]Lu-PSMA-617. Eur. J. Nucl. Med. Mol. Imaging.

[36] Ahmadzadehfar H., Rahbar K., Baum R.P., Seifert R., Kessel K., Boegemann M., Kulkarni H.R., Zhang J., Gerke C., Fimmers R. (2020). Prior Therapies as Prognostic Factors of Overall Survival in Metastatic Castration-Resistant Prostate Cancer Patients Treated with [177Lu]Lu-PSMA-617. A WARMTH Multicenter Study (the 617 Trial). Eur. J. Nucl. Med. Mol. Imaging.

[37] Braeuer A., Grubert L.S., Roll W., Schrader A.J., Schaefers M., Boegemann M., Rahbar K. (2017). 177Lu-PSMA-617 Radioligand Therapy and Outcome in Patients with Metastasized Castration-Resistant Prostate Cancer. Eur. J. Nucl. Med. Mol. Imaging.

[38] Kessel K., Seifert R., Schäfers M., Weckesser M., Schlack K., Boegemann M., Rahbar K. (2019). Second Line Chemotherapy and Visceral Metastases Are Associated with Poor Survival in Patients with MCRPC Receiving 177Lu-PSMA-617. Theranostics.

[39] Heck M.M., Tauber R., Schwaiger S., Retz M., D’Alessandria C., Maurer T., Gafita A., Wester H.J., Gschwend J.E., Weber W.A. (2019). Treatment Outcome, Toxicity, and Predictive Factors for Radioligand Therapy with 177Lu-PSMA-I&T in Metastatic Castration-Resistant Prostate Cancer (Figure Presented). Eur. Urol..

[40] Rahbar K., Bode A., Weckesser M., Avramovic N., Claesener M., Stegger L., Boegemann M. (2016). Radioligand Therapy with 177Lu-PSMA-617 as a Novel Therapeutic Option in Patients with Metastatic Castration Resistant Prostate Cancer. Clin. Nucl. Med..

[41] Rahbar K., Boegemann M., Yordanova A., Eveslage M., Schaefers M., Essler M., Ahmadzadehfar H. (2018). PSMA Targeted Radioligandtherapy in Metastatic Castration Resistant Prostate Cancer after Chemotherapy, Abiraterone and/or Enzalutamide. A Retrospective Analysis of Overall Survival. Eur. J. Nucl. Med. Mol. Imaging.

[42] Khreish F., Kochems N., Rosar F., Sabet A., Ries M., Maus S., Saar M., Bartholomä M., Ezziddin S. (2021). Response and Outcome of Liver Metastases in Patients with Metastatic Castration-Resistant Prostate Cancer (MCRPC) Undergoing 177Lu-PSMA-617 Radioligand Therapy. Eur. J. Nucl. Med. Mol. Imaging.

[43] Von Eyben F.E., Singh A., Zhang J., Nipsch K., Meyrick D., Lenzo N., Kairemo K., Joensuu T., Virgolini I., Soydal C. (2019). 177Lu-PSMA Radioligand Therapy of Predominant Lymph Node Metastatic Prostate Cancer. Oncotarget.

[44] Barber T.W., Singh A., Kulkarni H.R., Niepsch K., Billah B., Baum R.P. (2019). Clinical Outcomes of (177)Lu-PSMA Radioligand Therapy in Earlier and Later Phases of Metastatic Castration-Resistant Prostate Cancer Grouped by Previous Taxane Chemotherapy. J. Nucl. Med..

[45] Ahmadzadehfar H., Schlolaut S., Fimmers R., Yordanova A., Hirzebruch S., Schlenkhoff C., Gaertner F.C., Awang Z.H., Hauser S., Essler M. (2017). Predictors of Overall Survival in Metastatic Castration-Resistant Prostate Cancer Patients Receiving [177Lu]Lu-PSMA-617 Radioligand Therapy. Oncotarget.

[46] Rathke H., Holland-Letz T., Mier W., Flechsig P., Mavriopoulou E., Röhrich M., Kopka K., Hohenfellner M., Giesel F.L., Haberkorn U.A. (2019). Response Prediction of 177 Lu-PSMA-617 RLT Using PSA, Chromogranin A, and LDH. J. Nucl. Med..

[47] Ferdinandus J., Eppard E., Gaertner F.C., Kuerpig S., Fimmers R., Yordanova A., Hauser S., Feldmann G., Essler M., Ahmadzadehfar H. (2017). Predictors of Response to Radioligand Therapy of Metastatic Castrate-Resistant Prostate Cancer with 177Lu-PSMA-617. J. Nucl. Med..

[48] Violet J., Sandhu S., Iravani A., Ferdinandus J., Thang S.P., Kong G., Kumar A.R., Akhurst T., Pattison D.A., Beaulieu A. (2020). Long-Term Follow-up and Outcomes of Retreatment in an Expanded 50-Patient Single-Center Phase II Prospective Trial of 177Lu-PSMA-617 Theranostics in Metastatic Castration-Resistant Prostate Cancer. J. Nucl. Med..

[49] von Eyben F.E., Bauman G., von Eyben R., Rahbar K., Soydal C., Haug A.R., Virgolini I., Kulkarni H., Baum R., Paganelli G. (2020). Optimizing PSMA Radioligand Therapy for Patients with Metastatic Castration-Resistant Prostate Cancer. A Systematic Review and Meta-Analysis. Int. J. Mol. Sci..

[50] Kessel K., Seifert R., Weckesser M., Roll W., Humberg V., Schlack K., Bögemann M., Bernemann C., Rahbar K. (2020). Molecular Analysis of Circulating Tumor Cells of Metastatic Castration-Resistant Prostate Cancer Patients Receiving 177Lu-PSMA-617 Radioligand Therapy. Theranostics.

[51] Ahmadzadehfar H., Zimbelmann S., Yordanova A., Fimmers R., Kürpig S., Eppard E., Gaertner F.C., Wei X., Hauser S., Essler M. (2017). Radioligand Therapy of Metastatic Prostate Cancer Using 177Lu- PSMA-617 after Radiation Exposure to 223Ra-Dichloride. Oncotarget.

[52] Seifert R., Herrmann K., Kleesiek J., Schafers M.A., Shah V., Xu Z., Chabin G., Garbic S., Spottiswoode B., Rahbar K. (2020). Semi-Automatically Quantified Tumor Volume Using Ga-68-PSMA-11-PET as Biomarker for Survival in Patients with Advanced Prostate Cancer. J. Nucl. Med..

[53] Seifert R., Kessel K., Schlack K., Weber M., Herrmann K., Spanke M., Fendler W.P., Hadaschik B., Kleesiek J., Schaefers M. (2020). PSMA PET Total Tumor Volume Predicts Outcome of Patients with Advanced Prostate Cancer Receiving [177Lu]Lu-PSMA-617 Radioligand Therapy in a Bicentric Analysis. Eur. J. Nucl. Med. Mol. Imaging.

[54] Ferdinandus J., Violet J., Sandhu S., Hicks R.J., Ravi Kumar A.S., Iravani A., Kong G., Akhurst T., Thang S.P., Murphy D.G. (2020). Prognostic Biomarkers in Men with Metastatic Castration-Resistant Prostate Cancer Receiving [177Lu]-PSMA-617. Eur. J. Nucl. Med. Mol. Imaging.

[55] Violet J., Jackson P., Ferdinandus J., Sandhu S., Akhurst T., Iravani A., Kong G., Kumar A.R., Thang S.P., Eu P. (2019). Dosimetry of 177Lu-PSMA-617 in Metastatic Castration-Resistant Prostate Cancer: Correlations between Pretherapeutic Imaging and Whole-Body Tumor Dosimetry with Treatment Outcomes. J. Nucl. Med..

[56] Thang S.P., Violet J., Sandhu S., Iravani A., Akhurst T., Kong G., Ravi Kumar A., Murphy D.G., Williams S.G., Hicks R.J. (2019). Poor Outcomes for Patients with Metastatic Castration-Resistant Prostate Cancer with Low Prostate-Specific Membrane Antigen (PSMA) Expression Deemed Ineligible for 177Lu-Labelled PSMA Radioligand Therapy. Eur. Urol. Oncol..

[57] Han S., Woo S., Kim Y., Lee J.-L., Wibmer A.G., Schoder H., Ryu J.-S., Vargas H.A. (2021). Concordance between Response Assessment Using Prostate-Specific Membrane Antigen PET and Serum Prostate-Specific Antigen Levels after Systemic Treatment in Patients with Metastatic Castration Resistant Prostate Cancer: A Systematic Review and Meta-Analysi. Diagnostics.

[58] Calais J., Fendler W.P., Eiber M., Lassmann M., Dahlbom M., Esfandiari R., Gartmann J., Nguyen K., Thin P., Lok V. (2019). RESIST-PC Phase 2 Trial: 177Lu-PSMA-617 Radionuclide Therapy for Metastatic Castrate-Resistant Prostate Cancer. J. Clin. Oncol..

[59] Kratochwil C., Bruchertseifer F., Giesel F.L., Weis M., Verburg F.A., Mottaghy F., Kopka K., Apostolidis C., Haberkorn U., Morgenstern A. (2016). 225Ac-PSMA-617 for PSMA-Targeted Alpha-Radiation Therapy of Metastatic Castration-Resistant Prostate Cancer. J. Nucl. Med..

[60] Zacherl M.J., Gildehaus F.J., Mittlmeier L., Boening G., Gosewisch A., Wenter V., Schmidt-Hegemann N.-S., Belka C., Kretschmer A., Casuscelli J. (2020). First Clinical Results for PSMA Targeted Alpha Therapy Using 225 Ac-PSMA-I&T in Advanced MCRPC Patients. J. Nucl. Med..

[61] Yadav M.P., Ballal S., Sahoo R.K., Tripathi M., Seth A., Bal C. (2020). Efficacy and Safety of 225Ac-PSMA-617 Targeted Alpha Therapy in Metastatic Castration-Resistant Prostate Cancer Patients. Theranostics.

[62] Sathekge M., Bruchertseifer F., Knoesen O., Reyneke F., Lawal I., Lengana T., Davis C., Mahapane J., Corbett C., Vorster M. (2019). 225Ac-PSMA-617 in Chemotherapy-Naive Patients with Advanced Prostate Cancer: A Pilot Study. Eur. J. Nucl. Med. Mol. Imaging.

[63] Kratochwil C., Haberkorn U., Giesel F.L. (2020). 225Ac-PSMA-617 for Therapy of Prostate Cancer. Semin. Nucl. Med..

[64] Khreish F., Ebert N., Ries M., Maus S., Rosar F., Bohnenberger H., Stemler T., Saar M., Bartholomä M., Ezziddin S. (2020). 225Ac-PSMA-617/177Lu-PSMA-617 Tandem Therapy of Metastatic Castration-Resistant Prostate Cancer: Pilot Experience. Eur. J. Nucl. Med. Mol. Imaging.

